# Effects of Spent Mushroom Substrates and Compound Microorganism Preparation on the Growth Performance, Hematological Changes, and Intestinal Microbiota of Young Sika Deer

**DOI:** 10.3390/ani15162390

**Published:** 2025-08-14

**Authors:** Huali Shi, Tao Hou, Yundi Li, Sibo Cheng, Shukun Zhang, Min Wu, Chongshan Yuan, Aiwu Zhang

**Affiliations:** College of Animal Science and Technology, Jilin Agricultural University, Changchun 130118, China; 15584982356@163.com (H.S.); 18634611920@163.com (T.H.); 17785446215@163.com (Y.L.); 15754473057@163.com (S.C.); 18296078470@163.com (S.Z.); koo331500@163.com (M.W.)

**Keywords:** spent mushroom substrates, compound microorganism preparation, young sika deer, growth performance

## Abstract

Spent mushroom substrate (SMS) is abundant in nutrients, including polysaccharides, essential amino acids, dietary fiber (DF), and other bioactive compounds. The compound microorganism preparation (CMP), comprising multiple beneficial bacterial groups, is widely used as a feed additive to modulate intestinal microbiota and enhance nutrient absorption in animals. This study investigated the effects of supplementing young sika deer’s concentrated feed with SMS and CMP on total intestinal digestibility, growth performance, serum immune indicators, biochemical parameters, and intestinal microbial composition. Ninety animals were randomly assigned to three dietary treatments: control (basal diet), LE1 (basal diet + 5% SMS), and LE2 (basal diet + 5% SMS + 100 mg CMP/deer/day). Results showed that the LE2 group had significantly higher total weight gain (TWG) and average daily gain (ADG) than both the control group and the LE1 group. Levels of crude protein (CP), total protein (TP), albumin (ALB), blood glucose (GLU), and immunoglobulins (IgG, IgM, IgA) were significantly higher in the LE2 group than in the other groups, with LE1 also showing improvement over the control group. Additionally, ether extract (EE) and high-density lipoprotein (HDL) levels were found to be elevated in both the LE1 and LE2 groups when compared to the control group. In contrast, low-density lipoprotein (LDL) levels as well as urea nitrogen concentrations showed a significant increase in the LE1 group compared to both the control and LE2 groups. Notably, when compared to the control group, there was a significant reduction in the abundance of certain pathogenic bacteria within both the LE1 and LE2 groups; conversely, there was a substantial increase in the relative abundance of beneficial bacterial communities. These results demonstrate that combined SMS and CMP supplementation effectively improves growth performance, nutrient utilization, and intestinal health in young sika deer, suggesting its potential as a functional feed additive.

## Simple Summary

Spent mushroom substrate (SMS), a by-product of mushroom cultivation, is rich in various bioactive compounds that have the potential to promote animal growth and development. Compound microorganism preparation (CMP), comprising multiple probiotics, is recognized for its ability to enhance intestinal health. The combination of SMS and CMP may yield synergistic effects when used together. This study investigated the effects of dietary supplementation with CMP and SMS on apparent nutrient digestibility, growth performance, serum immune parameters, serum biochemical indicators, and the composition of the intestinal microbiota in young sika deer. The findings revealed that deer receiving both CMP and SMS exhibited the greatest weight gain, alongside improved digestibility of ether extract (EE) and crude protein (CP). In comparison to the control group and the SMS-only group, these deer demonstrated enhanced immune function as well as significant alterations in the relative abundance of specific bacterial genera within their intestinal microbiota. In conclusion, this study demonstrates that dietary supplementation with an appropriate ratio of CMP and SMS is both safe and effective in promoting growth and enhancing intestinal health in young sika deer. However, further research is necessary to ascertain the optimal dosage and proportion of CMP and SMS for achieving maximum efficacy.

## 1. Introduction

In the food processing industry, SMS by-products are commonly utilized for composting applications. These materials are rich in high-quality proteins, amino acids, phenolic compounds, and DF [1]. After fermentation or extraction processing, they demonstrate potent antioxidant and antibacterial activities [2,3]. Accumulating evidence indicates that SMS extracts and their bioactive components can modulate intestinal microbiota, immune responses, and nutrient digestion/absorption in animals [4,5]. As research on sika deer nutritional requirements advances, maintaining health status, meeting specific nutrient demands, and improving nutrient utilization efficiency remain key research priorities [6,7]. However, SMS application involves certain challenges. Proper SMS incorporation in feed formulations not only reduces production costs but also enhances immune competence, reduces oxidative stress, and alleviates inflammatory responses. Considering the relatively high fiber content of SMS [8] and the immature digestive systems of young deer, thorough safety assessments and optimal processing method development are essential for this target species.

Currently, aqueous extracts of SMS have been successfully applied in animal production systems [9]. However, research on the direct incorporation of SMS into conventional feed formulations remains scarce, primarily due to its complex matrix composition comprising residual fungal mycelium and partially degraded lignocellulose, which collectively account for its high DF content [8]. Recent studies have demonstrated that microbial fermentation can effectively reduce the DF content in SMS [10]. Notably, fermented SMS products have shown promising applications in livestock production, as evidenced by multiple studies [11,12].

CMP are bioactive substances that contain a diverse array of beneficial bacterial strains, including *Bacillus subtilis*, *Clostridium butyricum*, allicin, and *Enterococcus faecalis*. Experimental investigations into the co-fermentation of *Pleurotus eryngii* SMS with multiple microorganisms and enzymes have demonstrated that a treatment protocol involving the inoculation of *Bacillus subtilis*, *Lactobacillus acidophilus*, and *Saccharomyces cerevisiae*—alongside the supplementation of cellulase, xylanase, β-galactosidase, and urea—results in more favorable outcomes compared to using only cellulase, xylanase, and β-galactosidase. This approach has significantly accelerated the degradation rate of neutral detergent fiber while enhancing the nutrient content of the *Pleurotus eryngii* spent substrate [13]. Another study has shown that sawdust-based spent substrate from *Pleurotus ostreatus* can be recycled through probiotic fermentation for use as a feed supplement for post-weaning calves. Furthermore, this fermented sawdust-based substrate exhibits beneficial effects as an alternative to antibiotics and can act as a growth promoter for dairy cows [14]. The probiotics present in CMP have been found to modulate intestinal microbiota composition, improve digestive functions, and ferment DF within the gastrointestinal tract to facilitate nutrient absorption [15]. Additionally, phenolic compounds identified in fermented SMS may possess antioxidant properties along with free radical-scavenging capabilities [2]. These substances also contribute to reducing intestinal damage while maintaining epithelial cell integrity and functionality [16]. Consequently, incorporating probiotic-fermented SMS waste into animal feed presents significant application potential [17].

Therefore, this experiment assessed the effects of incorporating CMP and SMS into the concentrated feed for young sika deer on various growth performance indicators, total intestinal digestibility of macronutrients, serum biomarkers, immune-related serum indicators, and alterations in the intestinal microbiota community. The findings demonstrated that this feeding strategy significantly enhanced the growth performance of young sika deer as well as their apparent total digestibility of macronutrients. Additionally, it improved serum immune-related markers and facilitated beneficial modifications in the intestinal microbiota.

Through a comprehensive analysis of these parameters, this study further investigated the potential application of co-fermentation using SMS and CMP as functional feed ingredients in rearing young sika deer. It also examined their prospective value within sustainable ruminant farming practices. The results from this research provide a crucial foundation for optimizing both health and productivity in young sika deer while offering valuable insights for the rational utilization of SMS in ruminant agriculture.

## 2. Materials and Methods

### 2.1. Animals and Housing

In this study, a total of 90 healthy young sika deer (45 females and 45 males) were selected. The animals were approximately 150 ± 10 days of age, with an average body weight of 45.9 ± 1.4 kg. All sika deer were individually housed in separate enclosures at the breeding facility of Shuangyang Deer Industry Breeding Co., Ltd., located in the Shuangyang District, Changchun City, Jilin Province. The facility was maintained under strictly controlled environmental conditions.

The breeding management system was carefully designed to ensure continuous access to clean drinking water and sufficient roughage throughout the experimental period. Concentrated feed was administered to each group of sika deer at precisely 4:00 a.m. and 4:00 p.m. daily. This standardized feeding protocol was implemented to maintain consistent feeding schedules and promote optimal nutrient intake.

### 2.2. Experimental Design, Feed Composition, and Feeding Protocol

This study employed a completely randomized single-factor experimental design. A total of 90 young sika deer were randomly allocated into three experimental groups, with each group consisting of 30 individuals (one deer per replicate unit). The feeding trial was conducted over a period of 28 days. The SMS and CMP were provided by Jilin Agricultural University. Specifically, the SMS was air-dried and subsequently ground into a fine powder using a pulverizer. The CMP formulation primarily consisted of *Bacillus subtilis*, *Clostridium butyricum*, allicin, and *Enterococcus faecalis*. To address the specific nutritional needs of young sika deer, three distinct concentrated feed formulations were developed: the control group (basal diet), the LE1 group (basal diet + 5% SMS), and the LE2 group (basal diet + 5% SMS + 100 mg CMP/deer/day).

### 2.3. Body Weight Measurement

At the onset of the rearing period, the initial weight (IW) of each young sika deer was meticulously recorded, and the final weight (FW) was documented upon completion of the trial. All measurement data were systematically archived.

### 2.4. Fecal and Feed Sample Collection

During the final five days of the experimental period, fecal samples were collected daily via the total fecal collection method [18]. Concurrently, residual concentrate supplements and roughage from each group of young sika deer were meticulously collected. All samples (feces, supplements, and roughage) were oven-dried at 65 °C for at least 48 h until a constant weight was achieved, then ground through a 2 mm sieve and stored for subsequent laboratory analyses. Fresh fecal samples were additionally collected immediately post-defecation into 5 mL cryovials and preserved at −80 °C for in-depth gut microbiota characterization.

### 2.5. Blood Sample Collection and Analysis

On day 28 of the trial, ten sika deer per group were randomly selected. Each animal received an intramuscular injection of 1 mL xylazine hydrochloride for chemical restraint. Upon achieving surgical anesthesia (loss of palpebral reflex), jugular venipuncture was performed using 19G safety blood collection sets (Terumo® (Tokyo, Japan), BD Vacutainer® (Franklin Lakes, NJ, USA)) with 10 mL of whole blood transferred into serum separation tubes containing silica-based clot activator. Immediately after blood collection, each deer was administered 1 mL of idazoxan hydrochloride to reverse the effects of the anesthesia.

Upon collection of blood samples, serum was isolated by centrifugation at 4000× *g* for 10 min using an Eppendorf 5804 centrifuge (Eppendorf AG, Hamburg, Germany). The serum samples were then carefully collected, and serum parameters including TP, ALB, HDL, LDL, GLU, and urea were analyzed using a Hitachi 911 clinical chemistry analyzer (Hitachi High-Technologies Corporation, Tokyo, Japan).

To determine the concentrations of immunoglobulins IgA, IgM, and IgG in the serum, enzyme-linked immunosorbent assay (ELISA) kits [19] were employed. The specific kit models used were as follows: dpa-03-65 for IgA, dpa-03-66 for IgM, and dpa-03-64 for IgG. All kits were purchased from Beijing Deaoping Biotechnology Co., Ltd. (Beijing, China), with each immunoglobulin class corresponding to a specific kit according to the manufacturer’s protocols.

### 2.6. Growth Performance and Digestibility of Macro-Nutrients Measurement

The ADG and TWG of sika deer were calculated based on IW and FW. Specifically, TWG was defined as the difference between body weights measured on the first and last days of the experimental period, and ADG was obtained by dividing TWG by the trial duration (days). For dry matter (DM) analysis [20], concentrated feed, silage, and fecal samples were dried in a forced-air oven at 105 ± 2 °C until reaching constant mass (mass variation < 0.5 mg). Nitrogen (N) content was analyzed using a LECO FP-528N protein analyzer (LECO Corporation, St. Joseph, MI, USA). CP content was calculated as CP = N × 6.25 [21]. For EE determination [20], samples underwent Soxhlet extraction (Buchi B-811, Buchi Corporation, Flawil, Switzerland) with petroleum ether in a 75 °C water bath for 16 extraction cycles. Organic matter (OM) content [20] was determined by incinerating samples in a muffle furnace that ramped at 5 °C/min to 550 °C and held for 12 h. For calcium and phosphorus quantification [22], samples were microwave-digested with HNO_3_:HClO_4_ (4:1 *v*/*v*) and analyzed via ICP-MS (Agilent 7900, Agilent Technologies, Santa Clara, CA, USA).

### 2.7. Fecal Microbiota Analysis

Fecal DNA was extracted using the MagBead FastDNA Soil Kit (Cat. No. 116564384; MP Biomedicals, Santa Ana, CA, USA). The concentration of extracted DNA was measured with a Qubit 3.0 Fluorometer (Thermo Fisher Scientific, Waltham, MA, USA); molecular size was assessed by 0.8% agarose gel electrophoresis (120 V, 45 min); and DNA purity was quantified using a NanoDrop Spectrophotometer (Thermo Fisher Scientific, Waltham, MA, USA).

The hypervariable V3–V4 region of the bacterial 16S rRNA gene was selected for sequencing. PCR amplification was performed using specific primers targeting this region, with the following sequences: forward primer (F: 5′-ACTCCTACGGGAGGCAGCA-3′) and reverse primer (R: 5′-GGACTACHVGGGTWTCTAAT-3′). The thermal cycling protocol consisted of an initial denaturation at 98 °C for 5 min, followed by 25 cycles of denaturation at 98 °C for 30 s, annealing at 52 °C for 30 s, and extension at 72 °C for 45 s. A final extension step was carried out at 72 °C for 5 min, followed by indefinite holding at 12 °C. Purification of amplified products was achieved using the AxyPrep DNA Gel Extraction Kit (Axygen, Union City, NJ, USA).

For alpha diversity analysis, the QIIME 2 software (version 2023.9.1) was employed to calculate seven diversity indices for each sample: Chao1, Observed Features, Shannon, Simpson, Faith’s PD, Pielou’s evenness, and Good’s coverage. Box plots were created to visualize intergroup variations in these indices. Beta diversity was evaluated through unweighted UniFrac distance calculations performed with QIIME 2 (version 2023.9.1), followed by principal coordinate analysis (PCoA) and statistical testing implemented in R version 4.2.1.

Taxonomic composition was determined through amplicon sequence variant (ASV) classification coupled with SILVA 138.1 reference database alignment. The relative abundance of microbial taxa across phylum to genus levels was visualized via stacked bar plots generated with the ggplot2 package in R 4.3.1. To identify intergroup taxonomic differences, permutational multivariate analysis of variance was implemented in QIIME 2 (version 2023.9.1) with 999 permutations to quantify community structure dissimilarities. Additionally, the linear discriminant analysis effect size (LEfSe) method was utilized to identify biomarkers with differential abundance.

### 2.8. Statistical Analysis

Growth performance, macronutrient digestibility, serum biochemical parameters, and immune parameters were analyzed using SPSS Statistics 25.0 (IBM Corp., Armonk, NY, USA). Data were subjected to one-way analysis of variance (ANOVA) followed by post hoc comparisons with both the least significant difference (LSD) test and Duncan’s multiple range test. Results are presented as mean ± standard deviation. Statistical significance was defined at *p* < 0.05.

For microbiome data, bioinformatics analysis was performed using QIIME 2 (version 2023.9.1). Alpha diversity was assessed using multiple complementary indices: species richness was estimated using the Chao1 index [23] and Observed Species index; overall diversity was evaluated using the Shannon [24] and Simpson [25] indices; Faith’s Phylogenetic Diversity (Faith’s PD) [26] was applied to quantify phylogenetic diversity; Pielou’s evenness index [27] was used to assess the distribution evenness of taxa; and Good’s coverage index [28] was employed to estimate sequencing depth and library coverage. Beta diversity was analyzed using both weighted and unweighted UniFrac distances [29] to evaluate inter-sample differences in microbial community composition. Taxonomic annotation of sequencing data was carried out using the classify-sklearn naive Bayes plugin in QIIME 2 (version 2023.9.1). Subsequent classification was conducted by aligning sequences to the 99% similarity operational taxonomic units (OTUs) reference sequences from the Greengenes 13_8 database [30].

## 3. Results

### 3.1. Feed Nutrient Content

Table 1 summarizes the rough composition (on a dry matter basis) and nutritional specifications of the concentrated feed supplements. Table 2 lists the nutritional composition of the roughage raw materials, and Table 3 provides detailed nutritional characteristic data of the SMS.

### 3.2. Growth Performance and Apparent Digestibility

As delineated in Table 4, IW, FW, DM, and OM showed no statistically significant variation across the three treatment groups (*p* > 0.05). Notably, TWG, ADG, and CP parameters in the LE2 group demonstrated significant elevation compared to both the control and LE1 groups (*p* < 0.05). While EE content showed nonsignificant intergroup variation between LE1 and LE2, both experimental groups exhibited significantly higher EE values than controls (*p* < 0.05).

### 3.3. Serum Biochemical and Serum Immune Indicators

The results of serum biochemical and immune index assessments (Table 5) indicated that the levels of TP, ALB, IgA, IgM, IgG, and GLU in the LE2 group were significantly higher than those observed in both the control group and the LE1 group (*p* < 0.05). Additionally, the TP, ALB, IgA, IgM, IgG, and GLU levels in the LE1 group were also elevated compared to those in the control group (*p* < 0.05). However, the levels of urea and LDL in the LE1 group were significantly elevated compared to those in both the control group and the LE2 group (*p* < 0.05). There was no significant difference in HDL levels between the LE1 group and the LE2 group; however, both groups exhibited higher HDL levels than those observed in the control group (*p* < 0.05).

### 3.4. Intestinal Microbiota Community

**Figure 1:**
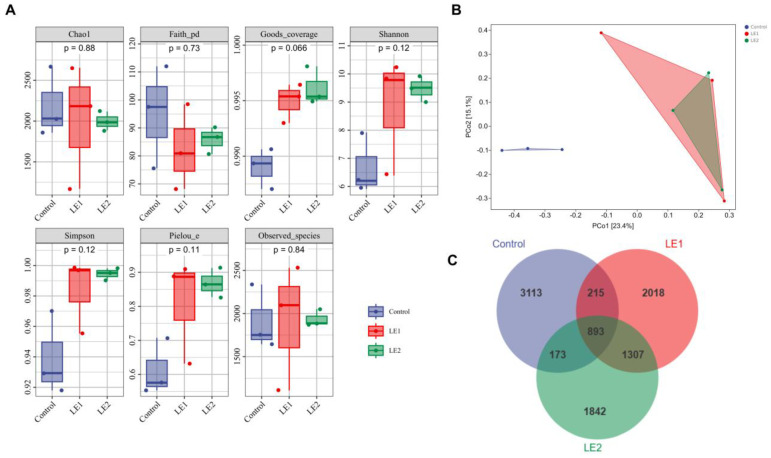
(**A**) Alpha diversity indices of the intestinal microbiota in young sika deer; (**B**) beta diversity analysis of the intestinal microbiota in young sika deer based on distance matrix and PCoA; (**C**) OTU-based venn diagram illustrating species differences and identification of marker species within the intestinal microbiota of young sika deer.

Alpha diversity assessment (Figure 1A) demonstrated significantly elevated Good’s coverage indices in LE1 and LE2 compared with controls (*p* < 0.05). Although Pielou’s evenness index, Simpson’s index, and Shannon’s index did not show significant increases in all three groups, these trends suggest that the evenness and diversity of the community had improved after the implementation of the intervention measures.

Beta diversity assessment (Figure 1B), conducted through Principal Coordinates Analysis (PCoA), revealed that the points representing the LE1 and LE2 groups were projected closer on the coordinate axes, indicating that the microbial community compositions of these two groups were similar. In contrast, the distances between the points of the control group and those of the LE1 and LE2 groups were larger, suggesting that the microbial community composition of the control group was different from that of both LE1 and LE2 groups. This indicates that SMS and CMP can alter the microbial community structure of young sika deer.

To identify shared and unique operational taxonomic units (OTUs) among the young sika deer groups, we performed a Venn diagram analysis based on OTU abundance data (Figure 1C). The results revealed a core of 893 OTUs shared across all three groups. The control group had 3113 unique OTUs, while the LE1 and LE2 groups contained 2018 and 1842 unique OTUs, respectively.

**Figure 2:**
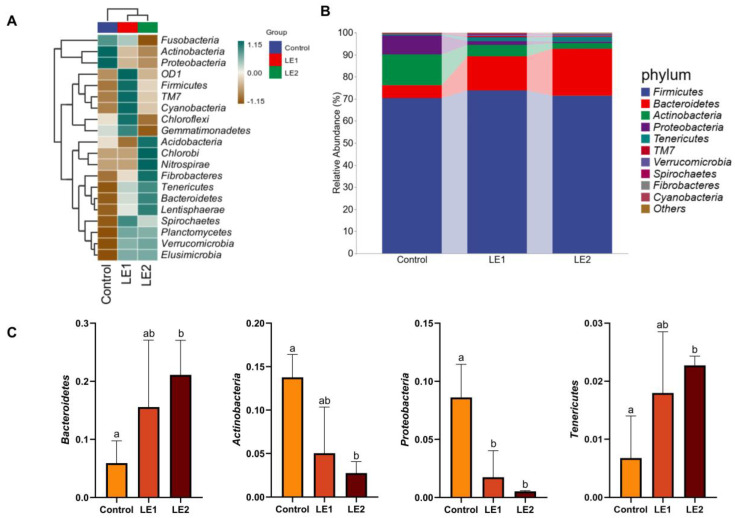
(**A**) Heatmap of species composition at the phylum level derived from the analysis of intestinal microbiota differences and identification of marker species in juvenile sika deer; (**B**) intestinal microbiota composition of juvenile sika deer at the phylum level; (**C**) comparative four-group analysis of microbiota at the phylum level. Values sharing different lowercase letters indicate statistically significant differences (*p* < 0.05).

To further clarify the inter-sample variations in species composition and to illustrate the trends in abundance distribution across samples, we constructed a heatmap of species composition utilizing the abundance data of the top 20 phyla ranked by their average abundance (Figure 2A). Furthermore, by integrating the observed group-level compositional differences derived from the heatmap with a statistical analysis of a feature table that has been filtered to remove singletons, we visualized the microbial composition at the phylum level (Figure 2B). The predominant bacterial phyla identified in the feces of young sika deer were *Firmicutes* (71.89%), *Bacteroidetes* (14.19%), *Actinobacteria* (7.18%), *Proteobacteria* (3.63%), *Tenericutes* (1.58%), *TM7* (0.41%), *Verrucomicrobia* (0.39%), *Spirochaetes* (0.27%), *Fibrobacteres* (0.18%), and *Cyanobacteria* (0.06%). Notably, the relative abundances of *Actinobacteria* and *Proteobacteria* were significantly diminished in both LE1 and LE2 groups when compared to the control group (*p* < 0.05). Furthermore, the LE2 group demonstrated a significantly greater abundance of *Tenericutes* and *Bacteroidetes* compared to the control group (*p* < 0.05) (Figure 2C).

**Figure 3:**
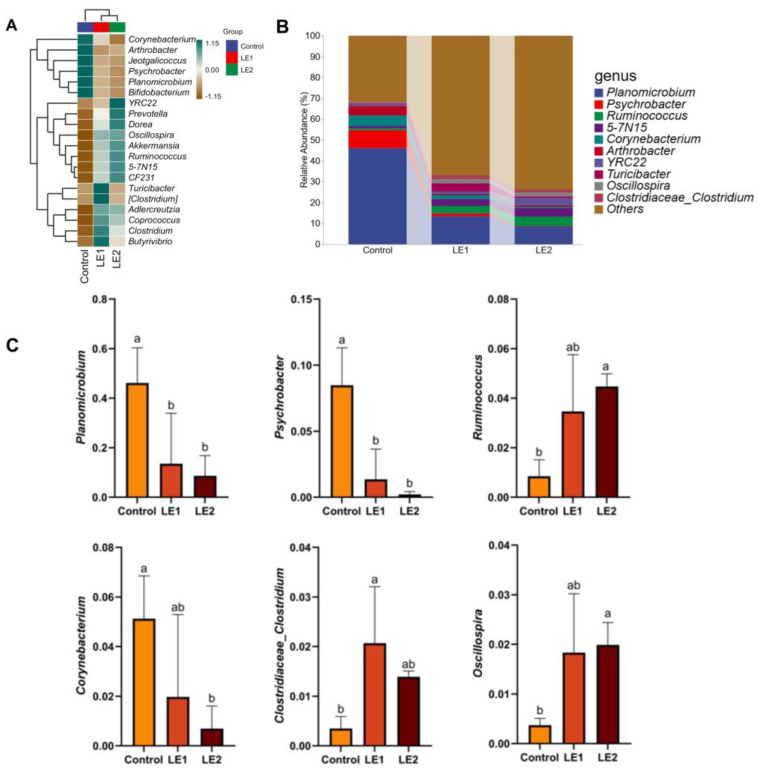
(**A**) Heatmap of genus-level species composition derived from the analysis of intestinal microbiota differences and identification of marker species in juvenile sika deer; (**B**) intestinal microbiota composition of juvenile sika deer at the genus level; (**C**) comparative six-group analysis of microbiota at the genus level. Values sharing different lowercase letters indicate statistically significant differences (*p* < 0.05).

We generated a heatmap illustrating species composition based on the abundance data of the top 20 genera ranked by average abundance (Figure 3A). By further integrating the intergroup compositional differences observed in the heatmap with statistical analyses of a feature table (filtered to exclude single-copy sequences), we visualized microbial composition at the genus level (Figure 3B). Comprehensive analysis of Figure 3A,B revealed the following predominant bacterial genera in young sika deer feces: *Planomicrobium* (22.76%), *Psychrobacter* (3.35%), *Ruminococcus* (2.93%), *5-7N15* (2.88%), *Corynebacterium* (2.52%), *Prevotella* (1.67%), *Arthrobacter* (1.92%), *YRC22* (1.83%), *Turicibacter* (1.71%), *Oscillospira* (1.40%), and *Clostridiaceae_Clostridium* (1.27%). The remaining genera collectively accounted for 57.42% of the total. Notably, the abundances of *Planomicrobium* and *Psychrobacter* were significantly reduced in both LE1 and LE2 groups compared to the control group (*p* < 0.05). In contrast, the LE2 group showed significantly higher abundances of *Ruminococcus* and *Oscillospira* relative to the control group (*p* < 0.05). Additionally, *Corynebacterium* abundance was markedly decreased in the LE2 group (*p* < 0.05), while *Clostridiaceae_Clostridium* abundance was significantly increased in the LE1 group compared to that of the control group (*p* < 0.05) (Figure 3C).

## 4. Discussion

SMS is recognized as a promising animal feed due to its richness in high-quality protein, amino acids, phenolic compounds, minerals, and DF [1]. However, the structural complexity and chemical stability of SMS-derived DF result in low degradation rates within the animal gastrointestinal tract, thereby limiting nutrient utilization efficiency [8]. Recent studies suggest that microbial consortia can effectively degrade SMS-DF, significantly improving its feed conversion efficiency and demonstrating substantial application potential [10]. The CMP used in this study contains multiple probiotic strains and functional microorganisms, which not only modulate intestinal microecological balance to enhance host immunity [31,32] but also act as fermentative agents for SMS, elevating its nutritional value through biotransformation processes [33]. While prior research has explored SMS applications in animal nutrition, the synergistic effects of SMS-CMP integration in sika deer diets remain unclear. Our previous studies independently evaluated the impacts of SMS on sika deer growth performance, macronutrient digestibility, blood biochemistry, and antler production [34,35]. Nevertheless, systematic data on how SMS-CMP co-administration influences the gut microbiota of young sika deer are critically lacking. To address this gap, the present study aims to (1) assess the effects of SMS-CMP supplementation on macronutrient apparent digestibility; and (2) analyze its impacts on serum biochemistry, immune parameters, and gut microbiota composition. These findings will provide a theoretical basis for optimizing SMS-CMP applications in ruminant nutrition.

In this experiment, the chemical composition of the concentrated supplementary feed was largely consistent across all treatment groups. However, minor variations in DF and CP content were observed in the feed supplemented with SMS. Previous studies have indicated that elevated DF levels may negatively affect animal weight gain [36], while microbial fermentation has been shown to significantly reduce DF content in SMS [10]. Although no significant differences were observed in IW and FW among the groups, the TWG and ADG of sika deer receiving both SMS and CMP were notably higher than those fed only SMS or the basal diet. According to Kim et al., microbially fermented SMS can enhance eye muscle area, improve growth performance and carcass traits in Korean beef cattle, and partially substitute for conventional roughage [37]. Therefore, we speculate that the addition of CMP helps to promote the effective degradation of DF during the rumen fermentation process of young sika deer. Additionally, this DF fraction remains within the range of being fermentable and degradable in the rumen.

No significant differences were detected in DM and OM content among the three experimental groups. However, when CMP was incorporated into the SMS-based diet, the apparent digestibility of CP was significantly improved compared to the group receiving only SMS and the control group. Although no statistically significant difference was found in EE apparent digestibility between the two experimental groups, both values were higher than those of the control group. This phenomenon can be explained by the following mechanisms: While increased DF content is generally negatively correlated with EE and CP digestibility [38], the combined inclusion of SMS and CMP in the basal diet may alter microbial metabolism via probiotics in CMP [39,40]. Elevated DF content in SMS promotes a metabolic shift toward glycolysis, thereby reducing protein degradation [41,42]. Additionally, probiotic fermentation activity from CMP in the rumen increases SMS protein content [17], which enhances lipid absorption [43].

In summary, based on experimental findings and observed differences in growth performance and digestibility, incorporating CMP not only improves nutrient absorption during SMS ruminal fermentation but also positively influences the overall growth performance of young sika deer.

It is critically important to closely monitor the health status of young deer throughout their growth and developmental stages. ALB and TP perform essential regulatory functions in the bloodstream, including binding to hormones, fatty acids, and bilirubin, modulating vascular permeability, and maintaining osmotic pressure [44,45]. Elevated levels of HDL are associated with reduced mortality risk from atherosclerosis and are characterized by anti-apoptotic, anti-thrombotic, and anti-infective effects [46,47,48]. Increases in IgG, IgA, and IgM reflect a significantly enhanced immune response [49]. In this study, compared with the control group, serum concentrations of TP, ALB, HDL, GLU, IgG, IgA, and IgM were elevated in both experimental groups following SMS supplementation. When SMS was combined with CMP, these parameters showed more favorable outcomes than those observed in the group receiving SMS alone. Furthermore, serum urea nitrogen levels in the CMP-supplemented group were significantly lower than in the group supplemented solely with SMS. Previous studies suggest that SMS possesses immunostimulatory properties [50], likely due to bioactive substances (e.g., polysaccharides with immunomodulatory activity) generated during ruminal fermentation [51,52].

The improved immune indicators observed in young sika deer administered both SMS and CMP suggest that the probiotic strains in CMP may synergistically enhance immune function [53,54]. Despite the structural complexity of SMS-derived polysaccharides [55,56], co-fermentation by ruminal microorganisms and CMP facilitates their breakdown in the large intestine, where they serve as carbon sources for intestinal microbiota [57]. Moreover, CMP supplementation increased dietary crude fiber degradation rates, which correlated with elevated serum GLU levels. These findings are consistent with existing research. Wang et al. [58] reported increased IgA, IgG, and IgM levels in fallow deer fed probiotics, while a study on forest musk deer demonstrated improved immune performance with probiotic supplementation [59]. Similarly, Wang et al. [60] observed reduced serum urea levels in sika deer fed fermented *Flammulina filiformis* residues. Collectively, these results indicate that SMS and CMP exert dual regulatory effects on serum biochemistry and immunity in sika deer, thereby promoting favorable physiological outcomes. Therefore, integrating SMS and CMP into immunomodulatory diets for sika deer holds significant potential for both scientific research and practical applications in animal nutrition management.

The diversity and richness of the intestinal microbiota play a crucial role in maintaining the host’s normal physiological functions [61]. To further investigate the effects of simultaneous supplementation with SMS and CMP on the intestinal microbiota, we employed high-throughput 16S rRNA sequencing to characterize the fecal microbial composition in young sika deer. Specifically, Alpha and Beta diversity indices provide a comprehensive assessment of intestinal microbiota diversity [62]. In this study, we first evaluated Alpha diversity, which quantifies species richness, diversity, and evenness within a local homogeneous habitat—also referred to as within-habitat diversity. Increases in both Simpson’s and Shannon’s indices indicated that the inclusion of SMS and CMP in the concentrated feed enhanced microbial richness and evenness [24,25]. Furthermore, the rise in Pielou’s evenness index suggested improved uniformity in the distribution of microbial taxa [27]. Meanwhile, Good’s coverage index reflected the adequacy and reliability of sequencing depth [28]. Our results showed that, although only Good’s coverage index exhibited statistically significant differences among the three experimental groups, all four α diversity indices increased when SMS was administered either alone or in combination with CMP. Beta diversity, which measures the dissimilarity in species composition across communities along environmental gradients or the rate of species turnover [29], revealed that the two experimental groups exhibited similar microbial communities, which were distinctly different from those of the control group. This indicates that both the combined and individual supplementation of SMS and CMP can alter the intestinal microbiota. Based on β diversity findings, we used Venn diagrams to analyze community structures, specifically identifying unique and shared OTUs across groups. Collectively, these findings support the conclusion that supplementing the diet of young sika deer with SMS and CMP positively influences intestinal microbiota diversity and composition.

To further compare the compositional differences in the intestinal microbiota at both the phylum and genus levels across experimental groups and to illustrate the species abundance distribution patterns among individual samples, we integrated species composition heatmap analysis with taxonomic profiling. At the phylum level, the analysis revealed that the intestinal microbiota of young deer was predominantly composed of *Firmicutes*, *Bacteroidetes*, *Actinobacteria*, *Proteobacteria*, and *Tenericutes*. *Firmicutes* and *Bacteroidetes* accounted for 71.89% and 14.19% of the total bacterial community, respectively. *Firmicutes* harbor genes involved in the fermentation of DF and can interact with the intestinal mucosa, thereby promoting energy homeostasis and supporting host health [63]. *Bacteroidetes* are capable of degrading complex polymers, thus facilitating nutrient digestion and absorption. This characteristic is particularly evident in herbivores consuming high-fiber diets, where *Bacteroidetes* are the most abundant phylum [64]. Notably, in the two experimental groups supplemented with SMS, the relative abundances of *Actinobacteria* and *Proteobacteria* in the intestinal microbiota of young sika deer were significantly lower than those in the control group, whereas the abundance of *Tenericutes* was markedly increased. Although no significant differences were observed in microbial community structure between the two experimental groups, the addition of CMP to SMS further reduced the abundances of *Actinobacteria* and *Proteobacteria*. *Proteobacteria* include various pathogenic bacteria with pro-inflammatory properties [65]; in contrast, certain members of *Tenericutes* possess the ability to degrade cellulose and participate in protein synthesis [66]. Bacteria within the phylum *Actinobacteria* can spontaneously produce organic acids—such as acetic acid, formic acid, lactic acid, and succinic acid—through glucose fermentation [67].

At the genus level, the intestinal microbiota of young deer is predominantly composed of *Planomicrobium*, *Ruminococcus*, *Psychrobacter*, *Corynebacterium,* and *Prevotella*. Although the abundances of *Planomicrobium* and *Psychrobacter* in the two experimental groups did not show statistically significant differences compared to the control group, both were numerically lower. Notably, compared with the control group, the abundances of *Ruminococcus, Clostridium* (within the family *Clostridiaceae*), and *Oscillospira* in the intestinal microbiota of young deer were significantly increased in both experimental groups, whereas the abundance of *Corynebacterium* was significantly reduced. *Ruminococcus*, a member of the phylum *Firmicutes*, is an important cellulose-degrading bacterium capable of producing cellulase complexes that break down cellulose [68]. *Clostridium* within the *Clostridiaceae* family is positively correlated with dietary protein content and protein digestibility and negatively correlated with fecal protein content [69]. The acidic by-products produced by this genus can lower intestinal pH, thereby promoting the growth of beneficial microbiota and inhibiting the proliferation of pathogenic bacteria [70]. *Oscillospira* has been shown to degrade polysaccharides and produce butyric acid—a metabolic process closely associated with body weight gain [71]. In contrast, certain species within the genus *Corynebacterium* are capable of producing exotoxins that may lead to ulcerative symptoms in affected animals [72]. Collectively, these findings suggest that the inclusion of SMS and CMP in the diet can positively modulate both nutrient digestion and immune function in sika deer by regulating the composition of the intestinal microbiota.

In conclusion, supplementing concentrated feed with CMP and SMS significantly improved nutrient digestibility. Furthermore, a synergistic interaction between the two additives was observed, leading to a marked enhancement in the health status of the intestinal microbiota in young deer. The findings of this study demonstrate that a daily supplementation regimen of 5% SMS and 100 mg CMP effectively promotes the growth, development, and immune function of young sika deer. Therefore, based on the current results, future research aimed at further optimizing the dosages of SMS and CMP is expected to yield even more pronounced beneficial effects.

## 5. Conclusions

SMS can be safely incorporated into the concentrated feed for young sika deer. The combined application of SMS and CMP significantly enhances the growth performance of young sika deer as well as the digestion and absorption of nutrients. This combination further strengthens immune function by optimizing the structure of the intestinal microbiota, thereby presenting an innovative strategy for the joint utilization of SMS and CMP.

## Figures and Tables

**Table 1:** Composition and nutrient levels of concentrated supplementary feed.

Item	Content (%)	Item	Content (%)
Corn	59.00	Moisture	13.51
Soybean Meal	30.00	Crude Protein	18.80
Corn Germ Meal	5.00	Crude Fat	2.52
Cane Molasses	1.00	Crude Ash	7.37
Sodium Chloride	2.00	Calcium	1.83
Limestone	1.00	Total Phosphorus	0.55
Minerals	1.00		
Vitamins	1.00		
Total	100.00		

**Table 2:** Nutrient composition of roughage (ensiled corn straws).

Item	Content (%)
Lactic Acid (%)	1.92
Crude Protein (%)	7.6
Crude Fat (%)	2.96
Crude Ash (%)	5.6
Acidic Detergent Fiber (ADF) (%)	19.55
Neutral Detergent Fiber (NDF) (%)	33.05

**Table 3:** Nutrient content of SMS (air-dried).

Item	Content (%; mg/kg)
Moisture (%)	10.53
Crude Protein (%)	13.72
Crude Fat (%)	11.63
Crude Ash (%)	4.65
Calcium (mg/kg)	62.55
Total Phosphorus (mg/kg)	193.05

**Table 4:** Effect of SMS and CMP on growth performance (kg) and apparent nutrient digestibility (%).

Items	Groups		
	Control	LE1	LE2
Initial Weight (IW)	43.00 ± 0.92	42.70 ± 1.71	41.43 ± 1.19
Final Weight (FW)	49.20 ± 0.56	48.93 ± 1.75	48.70 ± 0.87
Total Weight Gain (TWG)	6.20 ± 0.36 ^b^	6.23 ± 0.15 ^b^	7.27 ± 0.40 ^a^
Avg. Daily Gain (ADG)	0.22 ± 0.01 ^b^	0.22 ± 0.01 ^b^	0.26 ± 0.01 ^a^
Digestibility of DM (%)	71.02 ± 1.14	71.85 ± 1.22	72.97 ± 1.22
Digestibility of OM (%)	72.06 ± 1.85	71.74 ± 1.54	73.51 ± 2.47
Digestibility of EE (%)	64.37 ± 1.30 ^b^	66.81 ± 0.98 ^a^	66.70 ± 0.68 ^a^
Digestibility of CP (%)	69.83 ± 0.95 ^b^	71.07 ± 1.48 ^b^	74.54 ± 0.67 ^a^

Note: DM: dry matter; CP: crude protein; EE: ether extract; OM: organic matter. Means for different lowercase letters were significantly different (*p* < 0.05).

**Table 5:** Effect of SMS and CMP on serum biochemical index and immune globulin.

Items	Groups		
	Control	LE1	LE2
TP (g/L)	45.50 ± 0.53 ^c^	53.03 ± 2.90 ^b^	57.23 ± 0.81 ^a^
ALB (g/L)	24.40 ± 0.26 ^c^	26.87 ± 1.54 ^b^	30.57 ± 0.32 ^a^
HDL (mmol/L)	0.95 ± 0.01 ^b^	1.29 ± 0.09 ^a^	1.28 ± 0.05 ^a^
LDL (mmol/L)	0.18 ± 0.03 ^b^	0.22 ± 0.01 ^a^	0.17 ± 0.06 ^b^
GLU (mmol/L)	5.03 ± 0.09 ^c^	5.44 ± 0.22 ^b^	7.87 ± 0.17 ^a^
Urea (mmol/L)	9.62 ± 0.07 ^b^	10.08 ± 0.25 ^a^	9.27 ± 0.31 ^b^
IgG (g/L)	1.95 ± 0.13 ^c^	2.39 ± 0.70 ^b^	2.66 ± 0.42 ^a^
IgM (mg/L)	2.52 ± 0.47 ^c^	4.39 ± 0.15 ^b^	5.14 ± 0.29 ^a^
IgA (mg/L)	58.21 ± 4.16 ^c^	76.20 ± 5.73 ^b^	88.62 ± 3.09 ^a^

Note: TP: total protein; ALB: albumin; HDL: high-density lipoprotein; LDL: low-density lipoprotein; GLU: glucose; Urea: urea nitrogen; IgG: immunoglobulin G; IgM: immunoglobulin M; IgA: immunoglobulin A. Means for different lowercase letters were significantly different (*p* < 0.05).

## Data Availability

The data presented in this study are available upon request from the corresponding author.

## Abbreviations

SMS: spent mushroom substrate; CMP, compound microorganism preparations; LE1, with 5% SMS; LE2, with 5% SMS and fed 100 mg per day per animal of CMP; IW, initial weight; FW, final weight; TWG, total weight gain; ADG, average daily gain; DF, dietary fiber; DM, dry matter; CP, crude protein; EE, ether extract; OM, organic matter; TP, total protein; ALB, albumin; HDL, high-density lipoprotein; LDL, low-density lipoprotein; GLU, glucose; Urea, urea nitrogen; IgG, immunoglobulin G; IgM, immunoglobulin M; IgA, immunoglobulin A.
